# Editorial: Personalized Medicine for Urological Cancers: Targeting Cancer Metabolism

**DOI:** 10.3389/fonc.2022.862811

**Published:** 2022-03-03

**Authors:** Jennifer H. Gunter, Marianna Kruithof-de Julio, Eugenio Zoni

**Affiliations:** ^1^School of Biomedical Sciences, Faculty of Health, Institute of Health and Biomedical Innovation, Queensland University of Technology, Brisbane, QLD, Australia; ^2^Australian Prostate Cancer Research Centre-Queensland (APCRC-Q), Translational Research Institute, Queensland University of Technology, Woolloongabba, QLD, Australia; ^3^Urology Research Laboratory, Department for BioMedical Research (DBMR), University of Bern, Bern, Switzerland; ^4^Translational Organoid Resource, Department for BioMedical Research, University of Bern, Bern, Switzerland; ^5^Bern Center for Precision Medicine, Inselspital, University Hospital of Bern, Bern, Switzerland; ^6^Department of Urology, Inselspital, University Hospital of Bern, Bern, Switzerland

**Keywords:** metabolomics, prostate cancer, precision medicine, urological cancer, bladder cancer, kidney cancer

The key concept of personalized medicine is to identify the best treatment possible for a selected patient, in order to maximize therapeutic efficacy, reduce side effects, and minimize the risk of drug resistance development. To achieve this, it is fundamental to define effective cancer classifiers, which would allow appropriate patient stratification, minimizing overtreatment of indolent disease and avoiding delay in therapeutic treatments.

Sample availability, intra-tumor heterogeneity and the lack of established models for disease progression, have been the main challenges in deciphering the functional impact of genomic alterations on urological cancers, both in their common or rare forms. However, it has been possible to subtype urological cancers, mainly prostate and bladder, based on genomic alterations, while there is still a lack of knowledge of the metabolome. With the advances in metabolomics, the evaluation of metabolites has emerged as a strategy to identify new biomarkers. As discussed in the review by Singh R. et al. (Singh and Mills), there is a strong contrast between the capacity to sequence at high scale and decode genomic data in contrast with the metabolites that can be currently identified by mass spectrometry or other methods. When the technology can overcome this technical challenge, we will be able to prove the crosstalk between metabolic pathways and other cancer drivers and identify the causative connection of this interplay during disease onset and progression.

The aim of this Research Topic is to illustrate examples of personalized medicine for urological cancers, where assessment of metabolism can be used as strategy to refine disease diagnosis and patient prognosis.

## Bladder Cancer

As recently discussed by Minoli et al. the clinical guidelines and standard operating procedures for BLCa have limitations and physicians are often met with treatment failure and/or reoccurrence, indicating that the management of BLCa is complex and current classification systems do not depict the heterogeneity of this disease (1). Transurethral resection of bladder tumor (TURBT) combined with individualized intravesical chemotherapy or immunotherapy is recommended as the routine treatment model by the major international guidelines (Yang et al.). En bloc resection of bladder tumor (ERBT) has served as a valuable alternative technique that has attracted increasing interest among urologists globally. However, there is no robust clinical evidence that ERBT performs better than conventional resection in terms of disease progression. ERBT has the advantage of an intact tumor specimen containing detrusor muscle (DM) that can be used for accurate histopathological assessment by pathologists (Yang et al.). Additionally, such intact specimens are extremely valuable for characterization of tumor heterogeneity from a metabolic point of view in the context of personalized medicine. Similarly, a characterization of the metabolic effects of novel drugs is needed, to understand whether modulation at metabolic levels could be used as combinatorial approach with therapeutic agents. An example of FGFR3 mutant metastatic bladder cancer successfully treated with a combination of Anlotinib (2), a novel multitarget tyrosine kinase inhibitor, and Sintilimab (Zhang et al.), an antitumor PD-1 antibody, is illustrated in the case report by Cao et al. Another report presents the first case of urachal carcinoma treated with PD-1 antibody (Zheng et al.). At present, the most prominent issues for such rare tumors are the difficulty of obtaining drugs and the lack of late-stage clinical trials to guide therapeutic decisions. Matrix-assisted laser desorption/ionization (MALDI)-Orbitrap-mass spectrometry imaging (MSI) was recently used in a proof of principle study to showcase that such new methods can be used to identify biomarkers in a scenario where reliable diagnostic standards are not available (3).

## Renal Cell Carcinoma

The therapeutic scenario of metastatic renal cell cancer (mRCC) has noticeably increased in the last years, with the most recently introduced immunotherapies and tyrosine kinase inhibitors (TKI)-targeted therapies. Niu et al. have documented how the deletions in chromosomes 9 and 14, and the associated immunosuppressive microenvironment may indicate limited sensitivity to anti-PD-1/PD-L1 monotherapy for clear cell renal cell carcinoma patients with venous tumor thrombus. A new approach to predict clinical outcomes in mRCC patients treated with immune checkpoint inhibitors is the assessment of body composition, by measuring variables such as density of skeletal muscle (SM), subcutaneous fat, inter-muscular fat, and visceral fat. Martini et al. have proposed in this Research Topic that risk stratification using the body composition variables including total fat index may be prognostic and predictive of clinical outcomes in mRCC. The comprehensive review by Roberto et al. summarizes the latest clinical trials and provide guidance for overcoming the barriers to decision-making to offer a practical approach to the management of mRCC in daily clinical practice. The authors highlight the challenges to characterizing the renal neoplasia in all its complexity, as this would allow the lineage tracing of distant clones from a molecular point of view. Additionally, new challenges are still open such as patient stratification, which could also be achieved with metabolomics, treatment combination and timely intervention.

## Prostate Cancer

Lipids and their metabolism have recently reached the spotlight with accumulating functional evidence for promoting PCa onset, progression, and metastasis. This led to the definition of lipogenic signatures where a variety of intermediate of lipid metabolism are represented, ranging from bigger lipids (4) to intermediates of beta oxidation (5). In their review, Scaglia et al. discuss extensively how the metabolic machinery of lipid metabolism impact the tumor microenvironment and the therapeutic implications of targeting the lipogenic hubs, including dietary modulation. The concept that PCa progression is “a matter of fats” is supported by the research work of Faviana et al., which shows the potential of evaluating the expression of gastrin-releasing peptide receptor (GRPR) in PCa biopsy to improve our ability to detect PCa with low grades at the earliest phases of development. These findings support previous research showing the involvement of GRPR in adipocyte differentiation (6) and reinforce the notion that understanding which fat is good and which fat is bad for the prostate is crucial to deepen our understanding of the disease. In addition to lipids, the role of alternative nutrients and interactions with the tumor microenvironment are discussed in a review by Fidelito et al. which examines the challenges to metabolic therapies in prostate cancer, which may be met with the emergence of patient-derived organoids and the growing biobanks of PDX collections.

In conclusion, when technology will allow the definition of genomic-equivalent signatures in the cancer metabolome, significant progress will be achieved in the identification of features that are associated between genomics, metabolomics and epigenomics. Significant progress is being made in developing single-cell spatial methods for the detection of well-known metabolites, and mass spectrometry imaging is showing promising results. To capture metabolic information in real time in an operating theatre, there is a need for new devices/biomedical engineering, as the signals are dynamic/unstable compared to sequencing data where DNA is stable and technology needs to address those differences. As pointed out in the review by Singh and Mills, we can anticipate a future in which spatial genomic and metabolomic data are aligned to radiomic features and imaging to risk stratify patients and simultaneously inform treatment selection.

## Author Contributions

EZ assembled the editorial team, coordinated the work and wrote the manuscript. JG and MK-D participated in the editorial team and proof read the manuscript. All authors contributed to the article and approved the submitted version.

## Funding

EZ and MK received support from Krebsliga Schweiz under grant number KFS-4960-02-2020. EZ received support from Krebsliga Bern under grant number 41639. MK received support from SNSF Sinergia under grant number CRSII5_202297.

## Conflict of Interest

The authors declare that the research was conducted in the absence of any commercial or financial relationships that could be construed as a potential conflict of interest.

## Publisher’s Note

All claims expressed in this article are solely those of the authors and do not necessarily represent those of their affiliated organizations, or those of the publisher, the editors and the reviewers. Any product that may be evaluated in this article, or claim that may be made by its manufacturer, is not guaranteed or endorsed by the publisher.
